# Bioinformatics Analysis Identified Key Molecular Changes in Bladder Cancer Development and Recurrence

**DOI:** 10.1155/2019/3917982

**Published:** 2019-11-16

**Authors:** Qingke Chen, Jieping Hu, Jun Deng, Bin Fu, Ju Guo

**Affiliations:** Department of Urology, The First Affiliated Hospital of Nanchang University, Nanchang, Jiangxi 330006, China

## Abstract

*Background and Objectives*: Bladder cancer (BC) is a complex tumor associated with high recurrence and mortality. To discover key molecular changes in BC, we analyzed next-generation sequencing data of BC and surrounding tissue samples from clinical specimens. *Methods*. Gene expression profiling datasets of bladder cancer were analyzed online. The Database for Annotation, Visualization, and Integrated Discovery (DAVID, https://david.ncifcrf.gov/) was used to perform Gene Ontology (GO) functional and KEGG pathway enrichment analyses. Molecular Complex Detection (MCODE) in Cytoscape software (Cytoscape_v3.6.1) was applied to identify hub genes. Protein expression and survival data were downloaded from OncoLnc (http://www.oncolnc.org/). Gene expression data were obtained from the ONCOMINE website (https://www.oncomine.org/). *Results*. We identified 4211 differentially expressed genes (DEGs) by analysis of surrounding tissue vs. cancer tissue (SC analysis) and 410 DEGs by analysis of cancer tissue vs. recurrent tissue cluster (CR analysis). GO function analysis revealed enrichment of DEGs in genes related to the cytoplasm and nucleoplasm for both clusters, and KEGG pathway analysis showed enrichment of DEGs in the PI3K-Akt signaling pathway. We defined the 20 genes with the highest degree of connectivity as the hub genes. Cox regression revealed CCNB1, ESPL1, CENPM, BLM, and ASPM were related to overall survival. The expression levels of CCNB1, ESPL1, CENPM, BLM, and ASPM were 4.795-, 5.028-, 8.691-, 2.083-, and 3.725-fold higher in BC than the levels in normal tissues, respectively. *Conclusions*. The results suggested that the functions of CCNB1, ESPL1, CENPM, BLM, and ASPM may contribute to BC development and the functions of CCNB1, ESPL1, CENPM, and BLM may also contribute to BC recurrence.

## 1. Introduction

Bladder cancer (BC) is a common urogenital cancer, with an estimate of 80,470 new cases and 17,670 deaths in the United States in 2019 [1]. Bladder cancer patients are often diagnosed by cystoscopy for diagnostic testing prompted by haematuria. Approximately 80% of urinary bladder tumors are superficial papillary lesions but also can be multifocal and exhibit a tendency for recurrence: remaining tumors may invade the bladder wall and lead to distant metastases [2]. Treatment for BC includes transurethral resection of bladder tumor (TURBT), chemotherapy, or vaccine-based therapy directed to the bladder, cystectomy, radiotherapy, and chemotherapy [3]. However, BC is a complex disease associated with a high recurrence rate and high mortality, and its biology remains poorly understood [4].

There are several important risk factors for BC, such as cigarette smoking, occupational chemical exposure (especially to aromatic amines), water arsenic level, *Schistosoma haematobium* infection, and radiation therapy for pelvic malignancies [5]. Previous studies identified aspects of the molecular mechanism of BC development and recurrence. BC has been genetically associated with mutations of two genes, fibroblast growth factor receptor 3 (FGFR3, for low-grade, noninvasive papillary tumors), and tumor protein P53 (TP53, for high-grade, muscle-invasive tumors) [6]. Treatment with drugs targeting mutations in genes such as FGFR3, VEGF, signal transducer and activator of transcription 3, and CD24 has all shown preclinical activity [4]. Next-generation sequencing (NGS) has drastically increased the understanding of cancer processes including BC, and analyses of these data can provide insight into effective diagnostic and therapeutic BC treatments [7, 8].

There are significant BC molecular profiling data [9–12]. Researchers have explored screening of urine to detect DNA mutations as an alternative for urine cytology as a tool for the noninvasive detection and surveillance of BC [13]. Additionally, the analysis of frequently mutated genes in BC may suggest potential targets for personalized treatment and predict treatment response [8]. However, to date, it has been difficult to identify key genes related to BC from NGS data. To discover key molecules active in BC, we analyzed BC data from microarray experiments and NGS sequencing data of clinical specimens. Our results suggested CCNB1, ESPL1, CENPM, BLM, and ASPM may contribute to BC development and recurrence.

## 2. Materials and Methods

### 2.1. Online Data

The gene expression profiling datasets of bladder cancer were analyzed online (GEO; https://www.ncbi.nlm.nih.gov/geo/geo2r/?acc=GSE13507). 58 normal tissues surrounding cancer, 165 primary bladder cancer, and 23 recurrent samples were measured in this array.

### 2.2. Identifying Differentially Expressed Genes

To analyze the microarray data, we compared the gene expression between 58 normal tissues surrounding cancer and 165 primary bladder cancer samples to identify genes involved with tumorigenesis, and gene expression comparison between 165 primary bladder cancer and 23 recurrent samples was also performed to screen genes that promote tumor recurrence. Differentially expressed genes were screened by adjusted *p* value or *p* value and fold change (FC). For comparison between surrounding tissue and cancer tissue, differentially expressed genes were restricted by adjusted *p* value <0.05 and |FC| > 4, and we defined these genes cluster SC (surrounding tissue vs. cancer tissue). For comparison between cancer tissue and recurrent tissue, differentially expressed genes were restricted by *p* value <0.05 and |FC| > 2, and we defined these genes cluster CR (cancer tissue vs. recurrent tissue).

### 2.3. Merging Data

We proposed two methods to process the clusters SC and CR: (1) tumorigenesis and recurrence were promoted by the same genes or proteins, the overlap between SC and CR were the key genes, and overlap genes were analyzed to perform Gene Ontology and KEGG pathway analysis and retrieve interacting genes; (2) tumorigenesis and recurrence were contributed by different genes, we would find key genes from clusters SC and CR individually, and SC and CR genes were individually analyzed to perform Gene Ontology and KEGG pathway analysis and retrieve interacting genes. For method 1, Venny 2.1.0 (http://bioinfogp.cnb.csic.es/tools/venny/index.html) was used to identify overlapping differentially expressed genes between SC and CR. The upregulated and downregulated genes were measured, respectively.

### 2.4. Gene Ontology and KEGG Pathway Analysis

The Database for Annotation, Visualization, and Integrated Discovery (DAVID, https://david.ncifcrf.gov/) was used to perform Gene Ontology (GO) functional and KEGG pathway enrichment analyses. *p* < 0.05 was considered as statistically significant.

### 2.5. Retrieving Interacting Genes

Search Tool for the Retrieval of Interacting Genes (STRING) is an online tool (https://string-db.org) designed to integrate information by consolidating known and predicted protein-protein association data. Molecular Complex Detection (MCODE) in Cytoscape software (Cytoscape_v3.6.1) was applied to screen hub genes. All identified differentially expressed genes described above were analyzed. The top 20 hub genes with connection degree >10 were selected.

### 2.6. Survival Analysis

The protein expression and raw survival data were downloaded from OncoLnc (http://www.oncolnc.org/). Overall survival and disease-free survival were analyzed by Gene Expression Profiling Interactive Analysis (GEPIA, online website: http://gepia.cancer-pku.cn/detail.php?gene).

### 2.7. Gene Expression Data

Gene expression data were obtained from ONCOMINE website (https://www.oncomine.org/). Cancer type was restricted by bladder cancer, and the expressions of CCNB1, ESPL1, CENPM, BLM, ASPM, JUN, and CDK6 were obtained.

### 2.8. Statistical Analysis

Clinical information was analyzed by SPSS 18.0 (IBM Corporation, Armonk, NY). A Cox regression model was conducted to perform univariate and multivariate analyses. The gene expressions were analyzed by GraphPad Prism 7.0. *p* < 0.05 is considered to reveal a statistically significant difference.

## 3. Results

**Figure 1:**
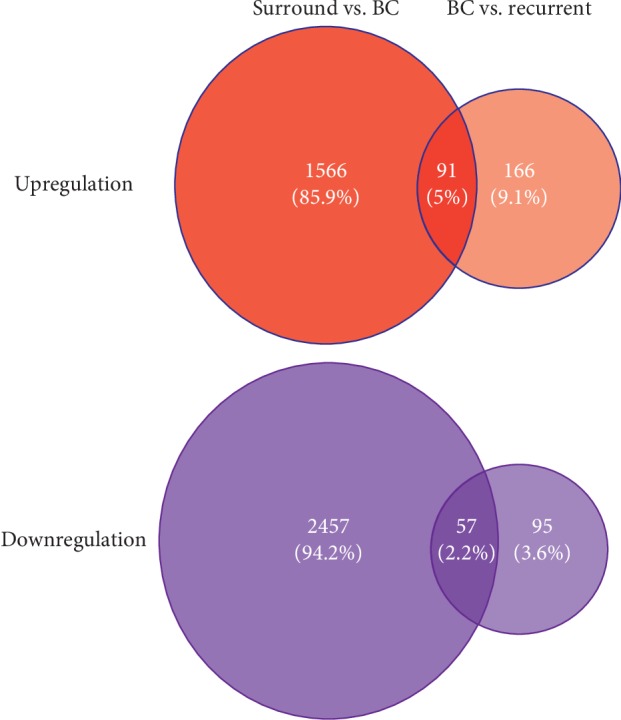
4211 differentially expressed genes (DEGs) were found by comparing 58 normal tissues surrounding cancer and 165 primary bladder cancer samples, and 410 DEGs were found by comparing 165 primary bladder cancer and 23 recurrent samples. There were 1657 and 258 upregulated DEGs and 2514 and 152 individually downregulated DEGs for each group. 91 and 57 overlap genes were found in up- and downregulation genes.

Analysis was performed using data for 58 normal tissues surrounding cancer, 165 primary bladder cancer samples, and 23 recurrent cancer samples. We identified 4211 differentially expressed genes (DEGs) by analysis of surrounding tissue vs. cancer tissue (SC analysis) and 410 DEGs by analysis of cancer tissue vs. recurrent tissue cluster (CR analysis). There were 1657 and 258 upregulated DEGs in cluster SC and cluster CR, respectively, and 2514 and 152 individually downregulated DEGs in cluster SC and cluster CR. A comparison of these sets of genes revealed 148 overlap genes, including 91 upregulated and 57 downregulated DEGs (Figure 1). We next analyzed these genes by performing two kinds of functional analysis.

### 3.1. Gene Ontology and KEGG Pathway Analysis

In the first analysis, the 91 upregulated and 57 downregulated genes that were differentially expressed in both the comparison of cancer and surrounding tissues and the comparison of cancer and recurrent cancer tissues were analyzed using the Database for Annotation, Visualization, and Integrated Discovery (DAVID; https://david.ncifcrf.gov/). Gene Ontology (GO) functional and KEGG pathway enrichment analyses were performed. GO function analysis revealed enrichment of these DEGs in functions related to the cytoplasm and nucleoplasm. There is an enrichment of genes involved with protein binding and protein kinase binding, regulating cell division, DNA replication, and cyclin-dependent protein serine/threonine kinase activity. KEGG pathway analysis indicated that the identified DEGs are mainly enriched in the PI3K-Akt signaling pathway, microRNAs related to cancer, and the cell cycle. The 15 most enriched classes based on GO function analysis and the eight most enriched KEGG pathways are listed in Table 1.

In the second analysis, we focused on the DEGs identified by the comparison of cancer and surrounding tissues or those identified by the comparison of cancer and recurrent cancer samples. Analysis of DEGs from the surrounding tissue vs. cancer tissue comparison should reflect key genes participating in tumorigenesis or bladder cancer development. GO function analysis of these genes found high enrichment of functions related to extracellular exosomes, extracellular space, and extracellular matrix. Protein binding, heparin binding, and integrin binding are the main functions of these genes, which participate in cell adhesion, extracellular matrix organization, and aging. KEGG pathway analysis indicated enrichment of these genes in HTLV-I infection, *Staphylococcus aureus* infection, and focal adhesion (Table 2). We next analyzed the DEGs identified by the comparison of cancer and recurrent cancer samples, which should include genes related to bladder cancer recurrence. GO function analysis revealed enrichment of these genes in functions related to the cytoplasm, cytosol, and nucleoplasm, and analysis of molecular function showed enrichment in protein binding. The most relevant enriched biological processes are angiogenesis and the G1/S transition of the mitotic cell cycle, and KEGG pathway analysis indicated enrichment of these genes in cancer pathways, the PI3K-Akt signaling pathway, and cell cycle (Table 2).

### 3.2. Hub Gene Analysis

**Figure 2:**
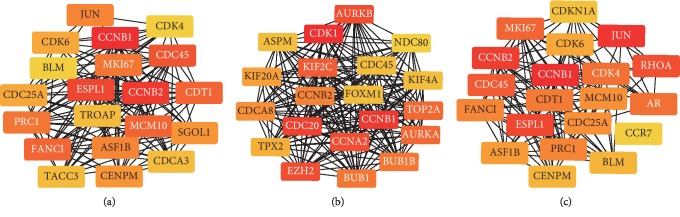
(a) According to method 1, 20 hub genes were discovered from overlapping differentially expressed genes between SC (surrounding tissue vs. cancer tissue) and CR (cancer tissue vs. recurrent tissue). (b) 20 hub genes were discovered from SC. (c) 20 hub genes were discovered from CR.

We used STRING for investigating and integrating interaction between proteins. Data were exported for further analysis by Cytoscape. We defined the top 20 genes with the highest degree of connectivity as the hub genes. For method 1, 20 hub genes are shown in Figure 2(a). Also, hub genes in clusters SC and CR are shown in Figures 2(b) and 2(c).

### 3.3. Clinical Analysis

**Figure 3:**
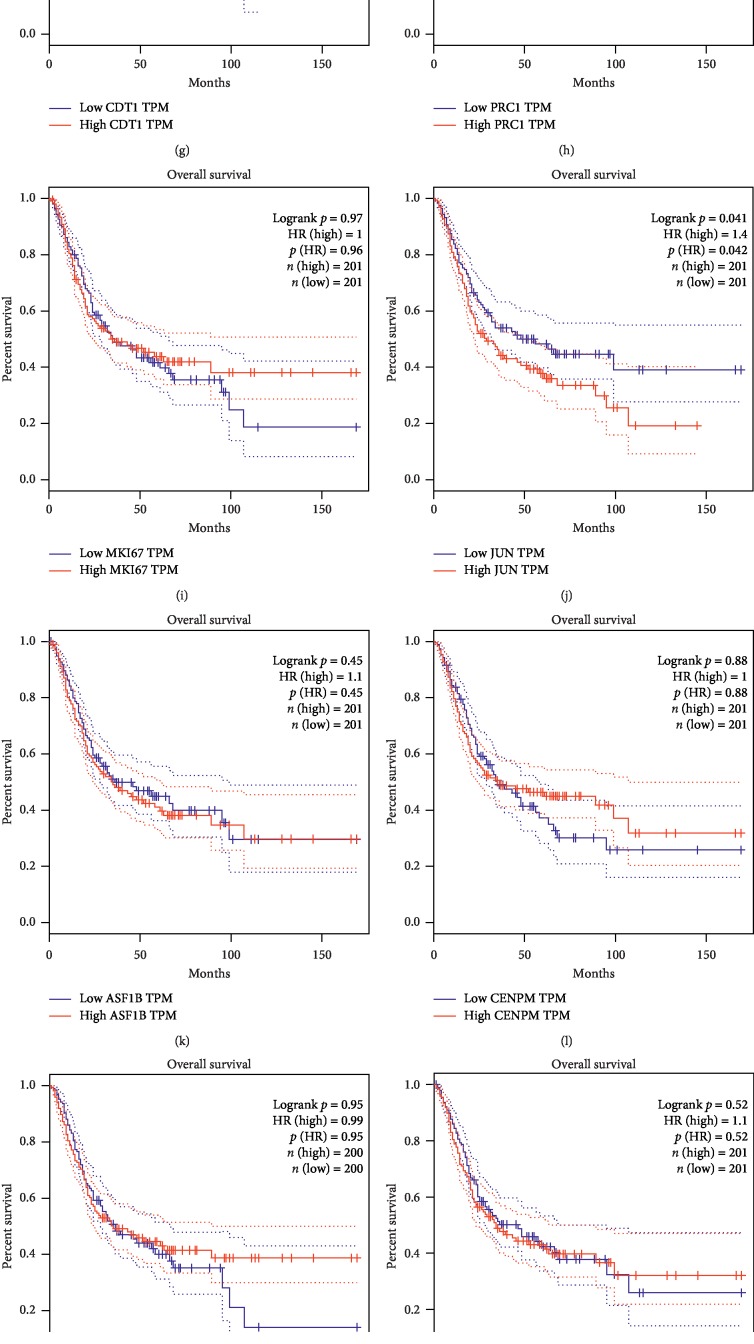
Overall survival analysis for 20 hub genes from method 1: (a) CCNB1, (b) CCNB2, (c) ESPL1, (d) CDC45, (e) FANCI, (f) MCM10, (g) CDT1, (h) PRC1, (i) MKI67, (j) JUN, (k) ASP1B, (l) CENPM, (m) SGOL1, (n) CDC25A, (o) CDK6, (p) CDCA3, (q) TACC3, (r) TROAP, (s) CDK4, and (t) BLM. For the 20 hub genes, only (j) JUN (HR = 1.4, *p*=0.041) and (o) CDK6 (HR = 1.5, *p*=0.013) showed statistical significance that higher expression patients indicated poor overall survival.

Kaplan–Meier analysis was performed for the identified hub genes using the DAVID website. We defined the 20 genes with the highest degree of connectivity as hub genes and determined hub genes for the SC comparison and for the CR comparison. For the 20 hub genes identified in the SC analysis, JUN and CDK6 were associated with the overall survival of bladder cancer patients (Figures 3(j) and 3(o)). High JUN expression increased the risk of death by 40% relative to low JUN expression (*p*=0.041), and high CDK6 expression increased the risk of death by 50% compared to low CDK6 expression (*p*=0.013). Overall survival analysis of other hub genes did not exhibit statistical significance for high and low expressions (Figures 3(a)–3(i), 3(k)–3(n), and 3(p)–3(t)).

We also determined 20 hub genes for the CR analysis. None of these hub genes were associated with overall survival (Supplement [Supplementary-material supplementary-material-1]). We next analyzed the hub genes and their association with disease-free survival (DFS) instead of overall survival. In this analysis, we found an association of CDK6 with DFS of bladder cancer patients (Supplement [Supplementary-material supplementary-material-1]).

We then downloaded the raw data from OncoLnc for further analysis. Cox regression revealed that CCNB1, ESPL1, CENPM, BLM, and ASPM are related to overall survival (Supplement [Supplementary-material supplementary-material-1]). Of these, CCNB1, ESPL1, CENPM, and BLM were identified as hub genes from cluster CR, and ASPM was identified as a hub gene from cluster SC (Supplement [Supplementary-material supplementary-material-1]).

### 3.4. Gene Expression in BC

**Figure 4:**
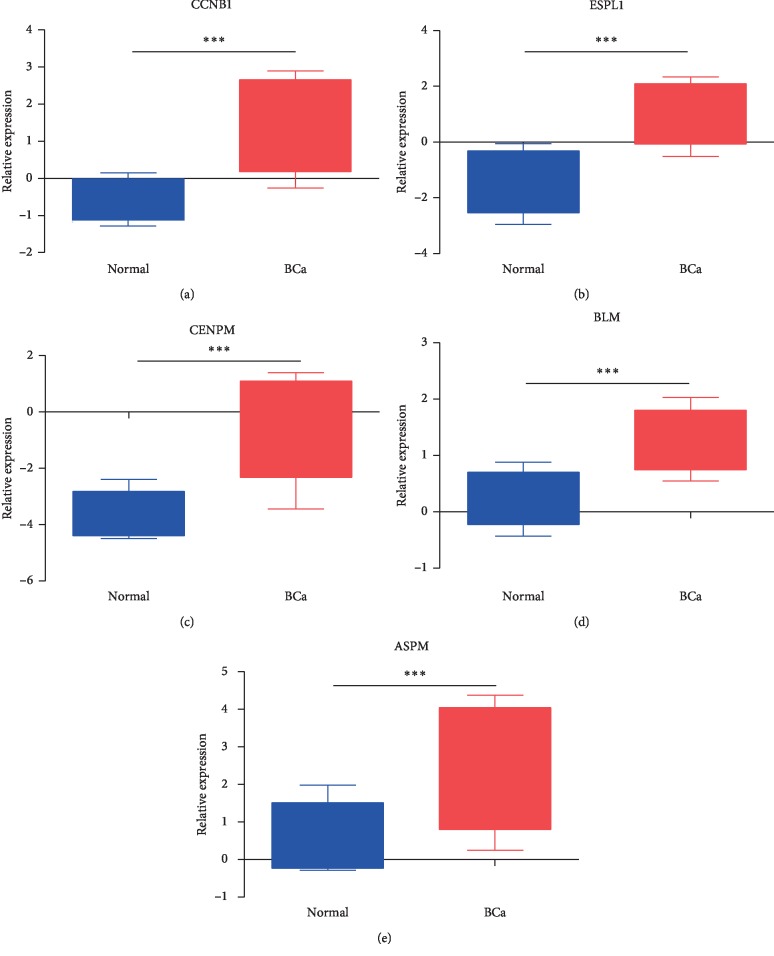
(a) CCNB1, (b) ESPL1, (c) CENPM, (d) BLM, and (e) ASPM were 4.795-, 5.028-, 8.691-, 2.083-, and 3.725-fold higher in BC than in normal tissues (*p* = 3.86*E*−13, 5.92*E*−20, 5.91*E*−26, 5.19*E*−14, and 2.56*E*−13, respectively).

The expressions of CCNB1, ESPL1, CENPM, BLM, ASPM, and two other genes (JUN and CDK6) associated with bladder cancer patient overall survival are shown in Figure 4 and Supplement [Supplementary-material supplementary-material-1]. The expression levels of CCNB1, ESPL1, CENPM, BLM, and ASPM were 4.795-, 5.028-, 8.691-, 2.083-, and 3.725-fold higher in BC than in normal tissues (*p* = 3.86*E*−13, 5.92*E*−20, 5.91*E*−26, 5.19*E*−14, and 2.56*E*−13). The expressions of JUN and CDK6 were not significantly different between BC and normal tissues (*p* = 0.639 and 0.466).

## 4. Discussion

In this analysis, we defined differentially expressed genes for the SC comparison of surrounding tissue vs. cancer tissue and for the CR comparison of cancer tissue vs. recurrent tissue and considered the identified DEGs contributing to BC development and contributing to BC recurrence, respectively. Genes found in both SC and CR analyses affect both BC development and recurrence, and key genes identified in either SC analysis or CR analysis but not in both analyses are genes that affect either BC development or recurrence, respectively. GO function analysis discovered DEGs are mainly enriched in cytoplasm and nucleoplasm for both clusters, and KEGG pathway analysis indicated high enrichment of DEGs in the PI3K-Akt signaling pathway. We found that CCNB1, ESPL1, CENPM, BLM, and ASPM may be associated with BC development, and CCNB1, ESPL1, CENPM, and BLM may be associated with BC recurrence. It was interesting that our analysis revealed four genes, CCNB1, ESPL1, CENPM, and BLM, which are associated with both BC development and recurrence. Although JUN and CDK6 were not associated with BC development or recurrence, they may be prognostic factors for overall survival (Figure 3(j), 3(o)). The *p* value was unadjusted for tumor recurrence, and without a correction for multiple tests, the results are meaningful but not conclusive for recurrent tumors.

Among the identified genes, we found CCNB1 was 4.8-fold more highly expressed in BC compared to the level in normal tissues (*p* = 3.86*E*−13). CCNB1 is an important cell cycle protein and is a key regulator of the G2/M checkpoint. High levels of CCNB1 usually lead to cell immortalization, resulting in aneuploidy, which contributes to chromosomal instability and is related to the aggressive nature of certain cancers [14]. The involvement of CCNB1 with BC was demonstrated previously [15–19]. Three bioinformatics analyses indicated that CCNB1 was a key gene in BC, consistent with our findings [17–19]; however, other hub genes reported previously such as KIF4A, TPX2, BUB1B, CDK1, ISG15, KIF15, RAD54L, and TRIP13 were not identified in our analysis. CCNB1 has been positively correlated with cell proliferation, invasion, and migration [20]. Gene expression profiling in 102 patients with non-muscle-invasive BC identified an association of CCNB1 with disease recurrence [16], and other analyses showed a positive correlation of CCNB1 with pathological stage and metastasis [20]. Cytological experiments may be required to confirm the function of CCNB1 in BC cells.

Our analysis discovered ESPL1 was expressed at a level 5.0-fold higher in BC than the level in normal tissues (*p* = 5.92*E*−20). ESPL1, also known as extra spindle poles-like 1 protein or separin, plays a central role in chromosome segregation by cleaving the cohesin complex at the onset of anaphase, and altered ESPL1 activity is correlated with aneuploidy and cancer [21]. Genomic analysis of transitional cell carcinoma (TCC) by both whole-genome and whole-exome sequencing of 99 individuals with TCC found frequent alterations in ESPL1 [22]. ESPL1 expression was negatively correlated with gastric adenocarcinoma pathologic stage progression, and the high expression of ESPL1 was significantly correlated with favorable outcomes [23]. In contrast, ESPL1 functions as an oncogene rather than as an antioncogene in breast cancer [24]. Further work is required to resolve the conflicting roles of ESPL1 in cancer and determine its function in BC.

CENPM was also identified as a key gene associated with BC. CENPM showed an 8.7-fold higher expression in BC compared to the levels in normal tissues (*p* = 5.91*E*−26). A study comparing the effects of garlic extracts and cisplatin for the treatment of BC identified 515 common anticancer genes, including CENPM. BC patients with low expression of CENPM showed significantly better progression-free survival than those with high expression of CENPM [25]. CENPM encodes centromere protein M, which is a component of the CENPA-NAC (nucleosome-associated) complex. The complex plays a central role in the assembly of kinetochore proteins, mitotic progression, and chromosome segregation [26]. Thus, we speculated that CENPM may be an important gene in BC development and recurrence.

BLM participates in DNA replication and repair and plays an important role in the maintenance of genome stability [27, 28]. Mutations altering BLM function are associated with highly elevated cancer susceptibility [29]. Its roles in BC are unknown, and our research suggests BLM function may be related to BC development and recurrence. The expression of BLM was 2.1-fold higher in BC than the level in normal tissues (*p* = 5.19*E*−14), but further research will be required to uncover the underlying mechanisms.

ASPM is the only gene that we found involved that was associated with BC development but not recurrence (Supplement [Supplementary-material supplementary-material-1]). ASPM exhibited a 3.7-fold higher expression level in BC than the level in normal tissues (*p* = 2.56*E*−13). Abnormal spindle-like microcephaly-associated protein is encoded by ASPM and is involved in mitotic spindle regulation and the coordination of mitotic processes [30]. Recently, another study reported significant overexpression of ASPM in bladder cancer that was associated with invasive pathological characteristics [31]. These results support our findings linking ASPM function to BC.

There are some limitations of this analysis that are worth noting. First, this research was based on data from a single gene array, so the inclusion of other expression data would strengthen the conclusions. Second, altered expression levels of these genes in BC have not been verified by biological methods, so additional experiments to knock down or overexpress these genes should be conducted. Finally, a major drawback of this study is insufficient evidence to suggest changes at the protein level, since the analysis was based only on mRNA expression data and protein interactions were predicted by STRING.

In conclusion, our study suggested CCNB1, ESPL1, CENPM, BLM, and ASPM may be associated with BC development, and CCNB1, ESPL1, CENPM, and BLM may be associated with BC recurrence. The functions of most of these candidate genes have not been the focus of previous studies of BC, and their functions in this cancer should be verified by in vivo and in vitro experiments.

## Figures and Tables

**Table 1:** Gene ontology and KEGG pathway analysis of differentially expressed genes according to method 1.

	Category	Term	Count	%	*p* value
*GO analysis*	GOTERM_CC_DIRECT	GO:0005654∼nucleoplasm	42	28.1879194630872	1.22*E* − 05
	GOTERM_MF_DIRECT	GO:0005515∼protein binding	92	61.744966442953	8.65*E* − 05
	GOTERM_BP_DIRECT	GO:0051301∼cell division	12	8.05369127516778	1.08*E* − 04
	GOTERM_BP_DIRECT	GO:0006260∼DNA replication	8	5.36912751677852	2.35*E* − 04
	GOTERM_CC_DIRECT	GO:0005737∼cytoplasm	60	40.2684563758389	2.68*E* − 04
	GOTERM_MF_DIRECT	GO:0019901∼protein kinase binding	11	7.38255033557047	7.94*E* − 04
	GOTERM_BP_DIRECT	GO:0000082∼G1/S transition of mitotic cell cycle	6	4.02684563758389	0.001302766851924
	GOTERM_CC_DIRECT	GO:0000922∼spindle pole	6	4.02684563758389	0.001440410268581
	GOTERM_MF_DIRECT	GO:0004693∼cyclin-dependent protein serine/threonine kinase activity	4	2.68456375838926	0.002393195271174
	GOTERM_BP_DIRECT	GO:0001706∼endoderm formation	3	2.01342281879194	0.003901253353245
	GOTERM_CC_DIRECT	GO:0005813∼centrosome	10	6.71140939597315	0.005184882974603
	GOTERM_CC_DIRECT	GO:0005739∼mitochondrion	20	13.4228187919463	0.005522684005422
	GOTERM_BP_DIRECT	GO:0051591∼response to cAMP	4	2.68456375838926	0.005751402857203
	GOTERM_BP_DIRECT	GO:0098609∼cell-cell adhesion	8	5.36912751677852	0.005854477268833
	GOTERM_CC_DIRECT	GO:0005829∼cytosol	38	25.5033557046979	0.007692882731082

*KEGG pathway*	KEGG_PATHWAY	hsa04110: cell cycle	7	4.6979865771812	0.00123536590764
	KEGG_PATHWAY	hsa05222: small cell lung cancer	5	3.35570469798657	0.009218413749072
	KEGG_PATHWAY	hsa04151: PI3K-Akt signaling pathway	9	6.04026845637583	0.018153887794995
	KEGG_PATHWAY	hsa05206: microRNAs in cancer	8	5.36912751677852	0.020570720541055
	KEGG_PATHWAY	hsa04115: p53 signaling pathway	4	2.68456375838926	0.02717134771579
	KEGG_PATHWAY	hsa04920: adipocytokine signaling pathway	4	2.68456375838926	0.030418432882382
	KEGG_PATHWAY	hsa04152: AMPK signaling pathway	5	3.35570469798657	0.031444562545736
	KEGG_PATHWAY	hsa04068: FoxO signaling pathway	5	3.35570469798657	0.04111192229316

BP: biological process; CC: cellular component; GO: gene ontology; MF: molecular function.

**Table 2:** Gene ontology and KEGG pathway analysis of differentially expressed genes in bladder cancer development and recurrence.

	Bladder cancer development					Bladder cancer recurrence				
	Category	Term	Count	%	*p* value	Category	Term	Count	%	*p* value
*GO analysis*	GOTERM_CC_DIRECT	GO:0070062∼extracellular exosome	331	25.5007704160246	6.61*E* − 26	GOTERM_MF_DIRECT	GO:0005515∼protein binding	233	57.2481572481572	5.48*E* − 07
	GOTERM_CC_DIRECT	GO:0031012∼+A1	69	5.31587057010785	2.26*E* − 19	GOTERM_CC_DIRECT	GO:0005737∼cytoplasm	152	37.3464373464373	1.25*E* − 06
	GOTERM_CC_DIRECT	GO:0005615∼extracellular space	164	12.6348228043143	1.98*E* − 13	GOTERM_CC_DIRECT	GO:0005654∼nucleoplasm	86	21.1302211302211	1.16*E* − 04
	GOTERM_BP_DIRECT	GO:0030198∼extracellular matrix organization	46	3.54391371340523	9.99*E* − 13	GOTERM_CC_DIRECT	GO:0016020∼membrane	70	17.1990171990172	2.74*E* − 04
	GOTERM_CC_DIRECT	GO:0005578∼proteinaceous extracellular matrix	54	4.16024653312788	1.67*E* − 12	GOTERM_CC_DIRECT	GO:0005829∼cytosol	96	23.5872235872235	4.19*E* − 04
	GOTERM_CC_DIRECT	GO:0005925∼focal adhesion	67	5.16178736517719	6.60*E* − 12	GOTERM_CC_DIRECT	GO:0070062∼extracellular exosome	83	20.3931203931203	6.83*E* − 04
	GOTERM_CC_DIRECT	GO:0009986∼cell surface	82	6.31741140215716	1.84*E* − 11	GOTERM_MF_DIRECT	GO:0004693∼cyclin-dependent protein serine/threonine kinase activity	6	1.47420147420147	7.00*E* − 04
	GOTERM_BP_DIRECT	GO:0007155∼cell adhesion	73	5.62403697996918	9.83*E* − 11	GOTERM_MF_DIRECT	GO:0030332∼cyclin binding	5	1.22850122850122	7.36*E* − 04
	GOTERM_CC_DIRECT	GO:0005576∼extracellular region	175	13.4822804314329	3.89*E* − 10	GOTERM_BP_DIRECT	GO:0048146∼positive regulation of fibroblast proliferation	7	1.71990171990172	8.99*E* − 04
	GOTERM_MF_DIRECT	GO:0005515∼protein binding	711	54.7765793528505	8.26*E* − 10	GOTERM_BP_DIRECT	GO:0000082∼G1/S transition of mitotic cell cycle	9	2.21130221130221	0.001364705455254
	GOTERM_MF_DIRECT	GO:0008201∼heparin binding	35	2.69645608628659	3.49*E* − 09	GOTERM_BP_DIRECT	GO:0048839∼inner ear development	6	1.47420147420147	0.00158628474829
	GOTERM_MF_DIRECT	GO:0005178∼integrin binding	27	2.08012326656394	8.57*E* − 09	GOTERM_BP_DIRECT	GO:0031581∼hemidesmosome assembly	4	0.982800982800982	0.001745599823436
	GOTERM_BP_DIRECT	GO:0048146∼positive regulation of fibroblast proliferation	19	1.46 *E* + 00	1.38*E* − 08	GOTERM_BP_DIRECT	GO:0051591∼response to cAMP	6	1.47420147420147	0.002664403703622
	GOTERM_BP_DIRECT	GO:0007568∼aging	33	2.54237288135593	1.37*E* − 07	GOTERM_BP_DIRECT	GO:0001525∼angiogenesis	13	3.19410319410319	0.002731528888927
	GOTERM_BP_DIRECT	GO:0060333∼interferon-gamma-mediated signaling pathway	20	1.54083204930662	2.89*E* − 07	GOTERM_CC_DIRECT	GO:0005615∼extracellular space	44	10.8108108108108	0.003130126302432

*KEGG pathway*	KEGG_PATHWAY	hsa05166: HTLV-I infection	49	3.77503852080123	1.54*E* − 08	KEGG_PATHWAY	hsa05200: pathways in cancer	22	5.4054054054054	0.001345594815917
	KEGG_PATHWAY	hsa05150: *Staphylococcus aureus* infection	20	1.54083204930662	1.67*E* − 08	KEGG_PATHWAY	hsa04151: PI3K-Akt signaling pathway	19	4.66830466830466	0.003848355864399
	KEGG_PATHWAY	hsa04510: focal adhesion	40	3.08166409861325	3.47*E* − 07	KEGG_PATHWAY	hsa04923: regulation of lipolysis in adipocytes	6	1.47420147420147	0.015090205294328
	KEGG_PATHWAY	hsa05323: rheumatoid arthritis	23	1.77195685670261	1.19*E* − 06	KEGG_PATHWAY	hsa04110: cell cycle	9	2.21130221130221	0.015736544810328
	KEGG_PATHWAY	hsa05416: viral myocarditis	18	1.38674884437596	1.41*E* − 06	KEGG_PATHWAY	hsa05222: small cell lung cancer	7	1.71990171990172	0.023482661953239
	KEGG_PATHWAY	hsa04514: cell adhesion molecules (CAMs)	30	2.31124807395993	2.37*E* − 06	KEGG_PATHWAY	hsa00010: glycolysis/gluconeogenesis	6	1.47420147420147	0.03037227765735
	KEGG_PATHWAY	hsa05310: asthma	12	0.924499229583975	1.17*E* − 05	KEGG_PATHWAY	hsa03320: PPAR signaling pathway	6	1.47420147420147	0.03037227765735
	KEGG_PATHWAY	hsa04110: cell cycle	26	2.00308166409861	1.49*E* − 05	KEGG_PATHWAY	hsa04610: complement and coagulation cascades	6	1.47420147420147	0.033914937801339
	KEGG_PATHWAY	hsa04610: complement and coagulation cascades	18	1.38674884437596	2.41*E* − 05	KEGG_PATHWAY	hsa05205: proteoglycans in cancer	11	2.7027027027027	0.036633631217231
	KEGG_PATHWAY	hsa04612: antigen processing and presentation	19	1.46379044684129	2.49*E* − 05	KEGG_PATHWAY	hsa04921: oxytocin signaling pathway	9	2.21130221130221	0.042657563729801

BP: biological process; CC: cellular component; GO: gene ontology; MF: molecular function.

## Data Availability

No data were used to support this study.

## Supplementary Materials

Supplementary Materials Supplement figure 1: overall survival analysis for 20 hub genes from cluster SC (surrounding tissue vs. cancer tissue) (A: CDK1; B. CCNB1; C: CDC20; D: CCNA2; E: AURKB; F: EZH2; G: KIF2C; H: AURKA; I: TOP2A; J: BUB1B; K: BUB1; L: CCNB2; M: CDCA8; N: KIF20A; O: TPX2; P: CDC45; Q: FOXM1; R: KIF4A; S: ASPM; T: NDC80). None of the hub genes showed statistical significance in comparing overall survival for high and low expression. Supplement figure 2: disease-free survival analysis for 20 hub genes from cluster CR (cancer tissue vs. recurrent tissue) (A: CCNB1; B: JUN; C: CCNB2; D: ESPL1; E: RHOA; F: CDC45; G: MKI67; H: CDK4; I: AR; J: CDT1; K: FANCI; L: PRC1; M: MCM10; N: CDC25A; O: ASF1B; P: CDK6; Q: BLM; R: CENPM; S: CDKN1A; T: CCR7). Only (P) CDK6 (HR = 1.4, *p* = 0.015) showed statistical significance that higher expression patients indicated poor disease-free survival. Supplement figure 3: the expressions of (A) JUN and (B) CDK6 were not significantly different between BC and normal tissues (*p* = 0.639 and 0.466). Supplement Table 1: Cox regression for hub genes in bladder cancer and clinical info.
