# Supplementary material for: Bioinformatics Analysis Identified Key Molecular Changes in Bladder Cancer Development and Recurrence
Source: Biomed Res Int. 2019 Nov 16;2019:3917982. doi: 10.1155/2019/3917982 (PMC6881748; doi:10.1155/2019/3917982)
Supplement: Supplementary Materials — Supplement figure 1: overall survival analysis for 20 hub genes from cluster SC (surrounding tissue vs. cancer tissue) (A: CDK1; B. CCNB1; C: CDC20; D: CCNA2; E: AURKB; F: EZH2; G: KIF2C; H: AURKA; I: TOP2A; J: BUB1B; K: BUB1; L: CCNB2; M: CDCA8; N: KIF20A; O: TPX2; P: CDC45; Q: FOXM1; R: KIF4A; S: ASPM; T: NDC80). None of the hub genes showed statistical significance in comparing overall survival for high and low expression. Supplement figure 2: disease-free survival analysis for 20 hub genes from cluster CR (cancer tissue vs. recurrent tissue) (A: CCNB1; B: JUN; C: CCNB2; D: ESPL1; E: RHOA; F: CDC45; G: MKI67; H: CDK4; I: AR; J: CDT1; K: FANCI; L: PRC1; M: MCM10; N: CDC25A; O: ASF1B; P: CDK6; Q: BLM; R: CENPM; S: CDKN1A; T: CCR7). Only (P) CDK6 (HR = 1.4, p = 0.015) showed statistical significance that higher expression patients indicated poor disease-free survival. Supplement figure 3: the expressions of (A) JUN and (B) CDK6 were not significantly different between BC and normal tissues (p = 0.639 and 0.466). Supplement Table 1: Cox regression for hub genes in bladder cancer and clinical info. [file 3917982.f1.zip › 3917982.f1/Supplement Table 1.docx]

| Supplement Table 1. Cox regression for hub genes in bladder cancer | | | | | | | |
| --- | --- | --- | --- | --- | --- | --- | --- |
| Method 1 | Gene | B | SE | Wald | df | Sig. | Exp(B) |
|  | CCNB1 | 0 | 0 | 4.344 | 1 | 0.037 | 1 |
|  | ESPL1 | 0.001 | 0 | 9.937 | 1 | 0.002 | 1.001 |
|  | CENPM | -0.001 | 0 | 3.937 | 1 | 0.047 | 0.999 |
|  | BLM | -0.001 | 0.001 | 6.373 | 1 | 0.012 | 0.999 |
| SC | ASPM | 0 | 0 | 19.548 | 1 | 0 | 1 |
| CR | CCNB1 | 0 | 0 | 4.344 | 1 | 0.037 | 1 |
|  | ESPL1 | 0.001 | 0 | 9.937 | 1 | 0.002 | 1.001 |
|  | BLM | -0.001 | 0.001 | 6.373 | 1 | 0.012 | 0.999 |
|  | CENPM | -0.001 | 0 | 3.937 | 1 | 0.047 | 0.999 |
